# Shading Beats Binocular Disparity in Depth from Luminance Gradients: Evidence against a Maximum Likelihood Principle for Cue Combination

**DOI:** 10.1371/journal.pone.0132658

**Published:** 2015-08-10

**Authors:** Chien-Chung Chen, Christopher William Tyler

**Affiliations:** 1 Department of Psychology, National Taiwan University, Taipei, Taiwan; 2 Center for Neurobiology and Cognitive Science, National Taiwan University, Taipei, Taiwan; 3 Smith-Kettlewell Eye Research Institute, San Francisco, California, United States of America; 4 Division of Optometry and Visual Science, School of Health Sciences, City University, London, United Kingdom; University College London, UNITED KINGDOM

## Abstract

Perceived depth is conveyed by multiple cues, including binocular disparity and luminance shading. Depth perception from luminance shading information depends on the perceptual assumption for the incident light, which has been shown to default to a diffuse illumination assumption. We focus on the case of sinusoidally corrugated surfaces to ask how shading and disparity cues combine defined by the joint luminance gradients and intrinsic disparity modulation that would occur in viewing the physical corrugation of a uniform surface under diffuse illumination. Such surfaces were simulated with a sinusoidal luminance modulation (0.26 or 1.8 *cy*/*deg*, contrast 20%-80%) modulated either in-phase or in opposite phase with a sinusoidal disparity of the same corrugation frequency, with disparity amplitudes ranging from 0’-20’. The observers’ task was to adjust the binocular disparity of a comparison random-dot stereogram surface to match the perceived depth of the joint luminance/disparity-modulated corrugation target. Regardless of target spatial frequency, the perceived target depth increased with the luminance contrast and depended on luminance phase but was largely unaffected by the luminance disparity modulation. These results validate the idea that human observers can use the diffuse illumination assumption to perceive depth from luminance gradients alone without making an assumption of light direction. For depth judgments with combined cues, the observers gave much greater weighting to the luminance shading than to the disparity modulation of the targets. The results were not well-fit by a Bayesian cue-combination model weighted in proportion to the variance of the measurements for each cue in isolation. Instead, they suggest that the visual system uses disjunctive mechanisms to process these two types of information rather than combining them according to their likelihood ratios.

## Introduction

When light reaches a surface, the shading pattern (or the luminance gradients) reflected from that surface to the eyes is jointly determined by the incident angle of the light and the local three-dimensional (3D) slant of the surface. Hence, the information regarding the 3D shape of an object is embedded in the luminance gradient of the two-dimensional (2D) image of that object [1,2,3]. The perception of shape-from-shading is thus an inverse problem for the recovery of the 3D shape of an object from the shading information in the 2D image.

However, if the direction of illumination is unknown, the solution to such a shape-from-shading problem is ambiguous or even indeterminate. As the luminance from a point on a surface is jointly determined by the direction of illumination and the surface slant, different combinations of illumination direction and surface slant can produce the same luminance distribution perceived by an observer [4,5,6]. For instance, a convex hemisphere illuminated from one direction has the same appearance as a concave hemisphere illuminated from the opposite direction [7,8,9].

One approach to the shape-from-shading problem is to find illumination-invariant properties in a scene [10,11]. For instance, Koenderink & van Doorn [12] suggested that shape-from-shading is based on the analysis of global luminance distributions such as the position of singularities of luminance and of the equiluminance contour. There are also studies on the illumination-invariant properties in the visual system that may help to determine shape from shading [13–17]. One example of this approach is demonstrated in hollow face illusion [14,15,18] in which the face is always perceived as convex regardless of the illumination even for the hollow mask of a face, suggesting that human observers employ a convex face assumption. However human observers in general do not show shape constancy under diverse illumination conditions [9, 19–22]. In addition, despite the strong demonstration of the hollow face illusion, there is evidence that face perception is strongly influenced by illumination direction [23]. The implication of these studies is that an illumination-invariant property of the image or the visual system cannot play a major role in shape-from-shading in general.

Another approach to the shape-from-shading problem is to find out how the visual system estimates the illumination direction and solves the inverse problem. An early approach by Pentland [4] proposed a model that assumed an isotropic distribution of surface orientations and that any bias in the luminance distribution of the image signals the illumination direction. This model requires the assumption of a unique light source from a definite direction. A very influential theory was put forward by Ramachandran [9,19] utilizing an observation by Brewster [7,24] and discussed by Gregory [8] to show that the human visual system resolves the shape-from-shading problem by making two assumptions about the illumination: 1) that there is a single light source illuminating the whole scene, and 2) that the light is shining from above (more precisely, with a slight top-left lighting direction [25,26]. With these two constraints, the visual system is often able to recover the 3D shape of the surface, in which a top-to-bottom luminance gradient from bright to dark suggests a convex surface while one from dark to bright suggests a concave surface [19].

The assumption of single light source, however, is often not tenable in the environment in general. Due to scattering, any scene containing the sky or a matt surface would have a diffused light component. Under diffuse illumination, such as in a cloudy day, the illumination comes from every direction and there is no single illumination direction. Hence, a model that assumes single-direction light sources cannot be applied to the case of shape perception under diffuse illumination.

Under diffuse illumination, the incident light from every direction has equal intensity. When from a diffuse source, the illumination at a point on a surface is the integral of all incident light at that point. Since the diffuse light intensity is uniform from all directions, the illumination at any point is a generalized cone of rays reaching that point through an aperture formed by the rest of the surface [27]. A point in a “valley”, which is by definition surrounded by neighboring “hills” has a reduced aperture for incident light, would receive less illumination and thus generally appear darker than a point on a “hill”, which is open to all incident light and thus appears brighter. Hence, the reflected light from a point on a Lambertian surface is approximately inversely related to the *depth* of that point and much of the shape of the surface can be recovered with the use of this “dark-is-deep” rule [6,28–31]. This analysis is different in principle from that under a single directional light source, in which the luminance is determined by the *orientation* of the surface related to the light source [1,2,32]. Langer & Bülthoff [6] showed that the observer can indeed discriminate between “hills” and “valleys” on a surface with this “dark-is-deep” rule. Schofield et al. [30] suggested that the shape-from-shading for a directional light source and for a diffuse light source are processed by parallel modules in the visual system.

Under normal viewing conditions, shading is not the only source of information for estimating the shape of a surface. A binocular observer can also assess the depth of a surface point through the binocular disparity of the shading information reflected from the surface. In particular, if a binocular observer views the physical corrugation of a uniform (Lambertian) surface under diffuse illumination, that surface will have intrinsic binocular disparity shifts of the peaks relative to the troughs, in addition to their corresponding luminance modulation from bright to dark. However, binocular disparity is based on the *difference* of luminance distributions between the left and right eye images, rather than on the luminance distribution itself [33,34]. Thus, it follows a different computational principle from the reconstruction of shape-from-shading in a single-image view.

In the present study, we are interested in how the visual system combines these two very different computational processes in estimating the shape of a surface. Previous work has shown an interaction between the visual processes for binocular disparity and shading from directional light source [35–39]. It is also reported that the presence of a diffusely illuminated corrugated surface can affect stereoscopic threshold [29]. Moreover, Likova & Tyler [40] investigated the perceived position of targets jointly determined by disparity and diffuse illumination cues. However, it is not known how the perceived depth is determined by binocular disparity and shading formed by diffuse illumination, which operates on a very different logic from that for shading from a focal light source.

**Fig 1:**
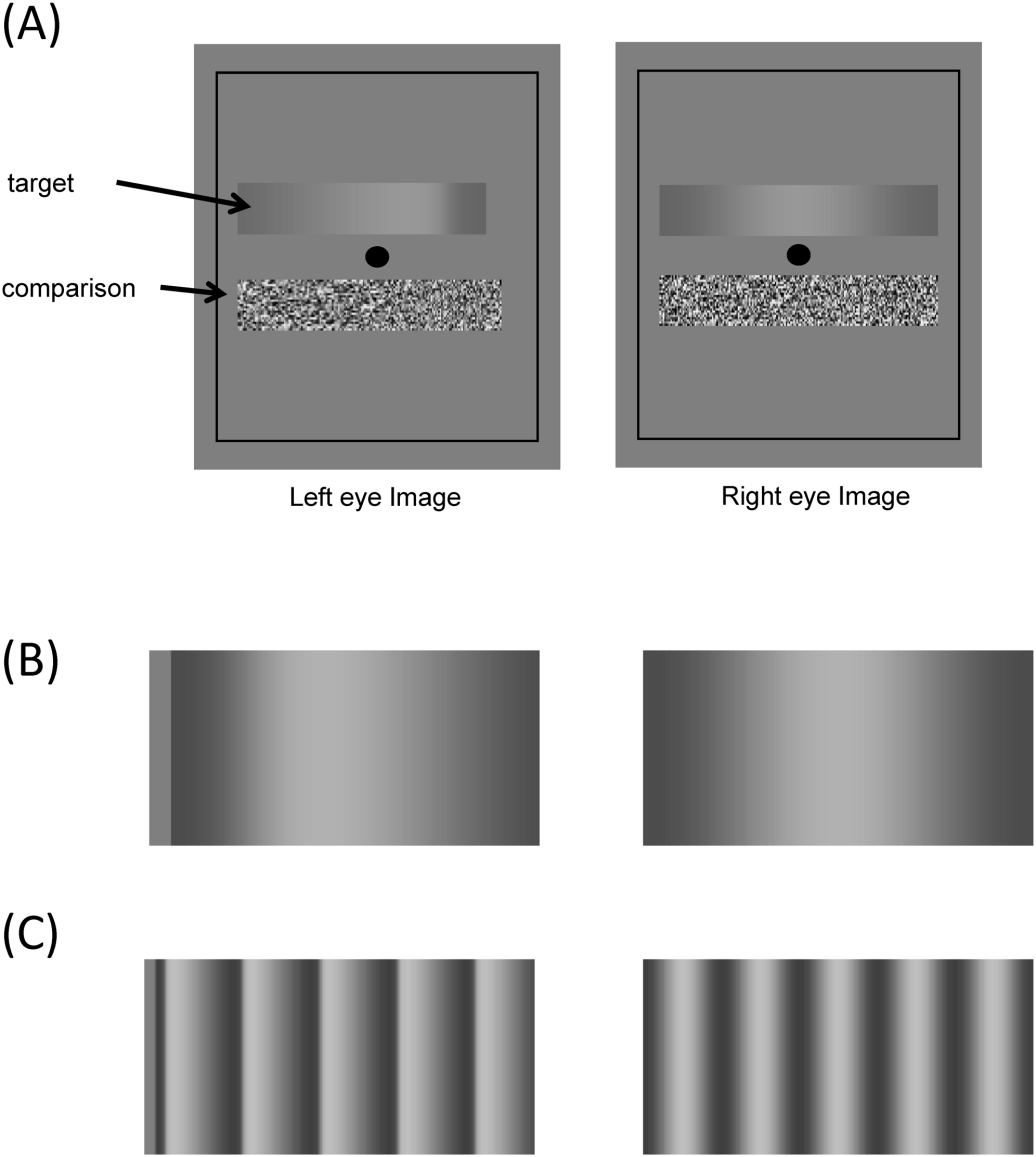
A. An example of the stimuli. Observers matched the perceived depth of the random-dot disparity comparison to that of the luminance+disparity defined depth of the target. B. Example of left-eye disparity shifts in the single raised cosine targets. C. Example of left-eye disparity shifts in the cosine wave targets.

We investigated this issue by measuring the perceived depth of sinusoidal luminance gratings with a disparity modulation appropriate to their generation by diffuse illumination of a corrugated sinusoidal surface. Notice that we did not attempt to capture the precise luminance profile of a physical sinusoidal surface illuminated with diffuse illumination and its own self reflections because of the complexity of such image generation and because human observers are generally insensitive to the second order subtleties of such luminance gradients. Instead we used a sinusoidal luminance profile combined with the corresponding sinusoidal disparity profile. Indeed, none of the participants were aware of any deviation from a sinusoidal depth profile for these stimuli under any conditions. Given the dark-is-deep rule, the part of the grating with lower luminance should be perceived as further away from an observer than the part with higher luminance [28]. The sinusoidal luminance modulation was itself modulated differentially between the two eyes to provide a disparity cue to the same corrugation profile, but for a range of different positive and negative disparity amplitudes (see Fig 1). For example, the luminance peaks were shifted to the left in one eye while the luminance troughs were shifted to the right, with corresponding differences in the luminance gradients between them, to provide the disparity cue that would be obtained from viewing a physically corrugated surface that was diffusely illuminated. By observing the change of perceived depth with different combinations of luminance contrast and disparity modulation, we can establish how the visual system integrates these two types of information in determining the shape of such a surface.

## Methods

### Apparatus

The sinusoidal corrugation stimuli were presented on the 21-inch CRT monitor with 1920 (H) ×1440 (V) spatial resolution and 75 Hz refresh rate controlled by a Quad-Core MacPro computer. The viewing field for each eye (half of the display width) was 9.9° (H) by 14.6° (V). Observers viewed the monitor at a distance of 112 cm, at which one pixel on the screen subtended 0.01°×0.01°. The left and the right sides of the screen were masked by a black divider to avoid cross-talk. Observers viewed stimuli through a four-mirror stereoscope in a dark, quiet room. The monitor input-output intensity function was calibrated to full linearity (within 0.1% at all 256 luminance levels) with a LightMouse photometer [41]. The observer’s head was supported by a chin rest. The experimental control and the stimulus generation were written in MATLAB with the Psychophysics Toolbox [42].

### Stimuli

Fig 1 shows an example of the stimuli. Both the left and the right images contained a fixation point (0.05° × 0.05°) at the center of the display, a black rectangular surround (2.2° (H) × 1.75°(V) with line width 0.03°) to help establish binocular fusion, a target 1.8° (H) × 0.45°(V) whose center was 0.32° above the fixation, and a matchable stimulus of the same size whose center was 0.32° below the fixation

The simulated surface in our stimuli had a sinusoidal luminance profile:

$$\text{L}\left(\text{x}\right)\;=\;L_{0}\;+\text{ C }\times \text{ cos}\left(2\pi \text{fx}\right)$$

where L(x) was the luminance at point x; *L*_0_ was the mean luminance of the display 27.6 cd/*m* ^2^; C was the contrast; and f was the spatial frequency of the luminance profile. The luminance contrast C was ±20% and ±80%. The positive contrast was defined as having the luminance at the fixation position closer than average and the negative, farther. The spatial frequency was either 1.8 *cy*/*deg* to simulate a corrugated cosine wave surface or 0.26 *cy*/*deg* to simulate a single raised-cosine bulge (C > 0) or a dip (C <0).

The disparity modulation of the target also had a sinusoidal profile:

$$\text{d}\left(\text{x}\right)\;=\;d_{c}\;\times \text{ cos}\left(2\pi \text{fx}\right)$$

where d(x) was the disparity at point x and *d*_*c*_ was the maximum disparity in the stimulus. The spatial frequency of disparity modulation was the same as that for the luminance modulation. The disparity *d*_*c*_ ranged from -20 to 20 arcmin in this study. Positive *d*_*c*_ meant the surface pointed toward the observer and the negative *d*_*c*_, away from the observer, with the sign assignment of the disparity consistent with that of luminance contrast.

The match stimuli were random-dot stereograms with a uniform dot distribution. Each dot was a 0.02° × 0.02° square. Tyler [28] showed that, due to the compensating nonlinearities of the luminance relative to the z-axis height of a 3D surface under diffuse illumination and the compressive nonlinearity of human luminance perception, the perceived height of points on a corrugated surface under diffuse illumination is well-approximated by a linear proportionality to its luminance. Hence, the matching stimulus was given the same sinusoidal spatial profile as the target disparity modulation except for its amplitude, which was the adjustment parameter.

### Procedures

Throughout the experiment, the fixation and the surrounding squares were present on the screen to help the observer to maintain fusion of the left and right eye images. On each trial, both the target and the match stimuli appeared 100 ms after a tone. The observer then pressed one of the two keys on a keypad to adjust the disparity of the matching stimulus until the perceived depth of the match and the target appeared equal, with both the target and the match remaining on screen throughout the trial. When satisfied with the match, the observer then pressed another key to finish the trial. The luminance contrast and disparity of the target and the initial disparity of the match were randomized for each trial.

### Participants

Five observers participated in the experiment. All were naïve to the purpose of this study and had normal or corrected-to-normal vision acuity (20/20). Before the experiment, observers were shown some of the random-dot match stimuli used in the experiment to verify that they were able to identify the 3D shape implied by the stimuli. All observers provided written consent and were financially compensated for their time. The use of human observers was approved by the Research Ethics Committee of the National Taiwan University Hospital (201210026RIC) and followed the guidelines specified in the Declaration of Helsinki.

## Results

**Fig 2:**
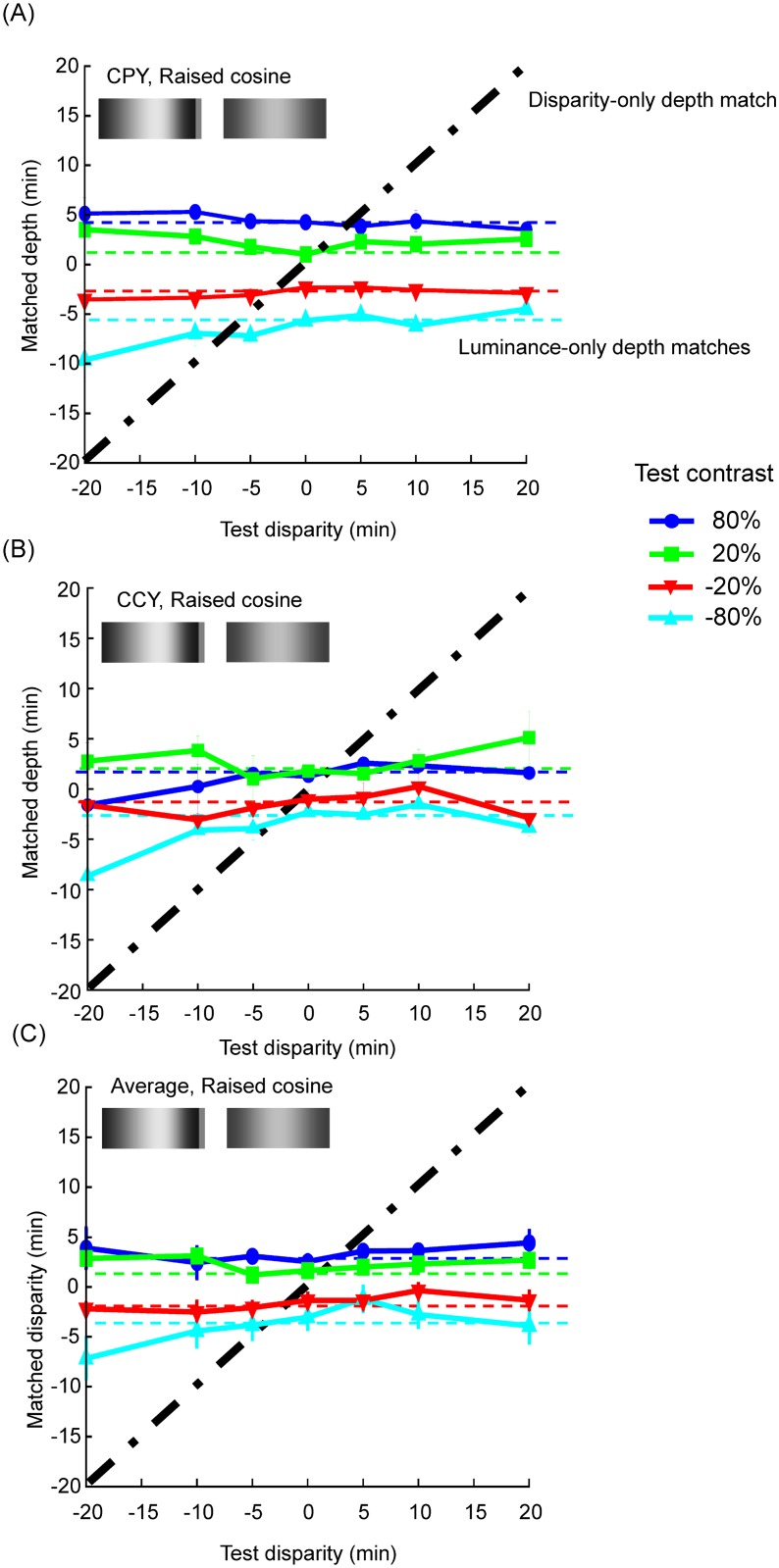
Matched depth as a function of the test disparity for luminance gradients in two representative observers (A) and (B), and the average across five observers (C) in the single raised cosine (0.26 *cy*/*deg* test spatial frequency) condition. Horizontal dashed lines denote the matched disparity for zero test disparity at the corresponding test luminance contrast. The diagonal dash-dot lines show the predictions for depth matches as a function of target disparity only. The error bars represent one standard error of the means.

**Fig 3:**
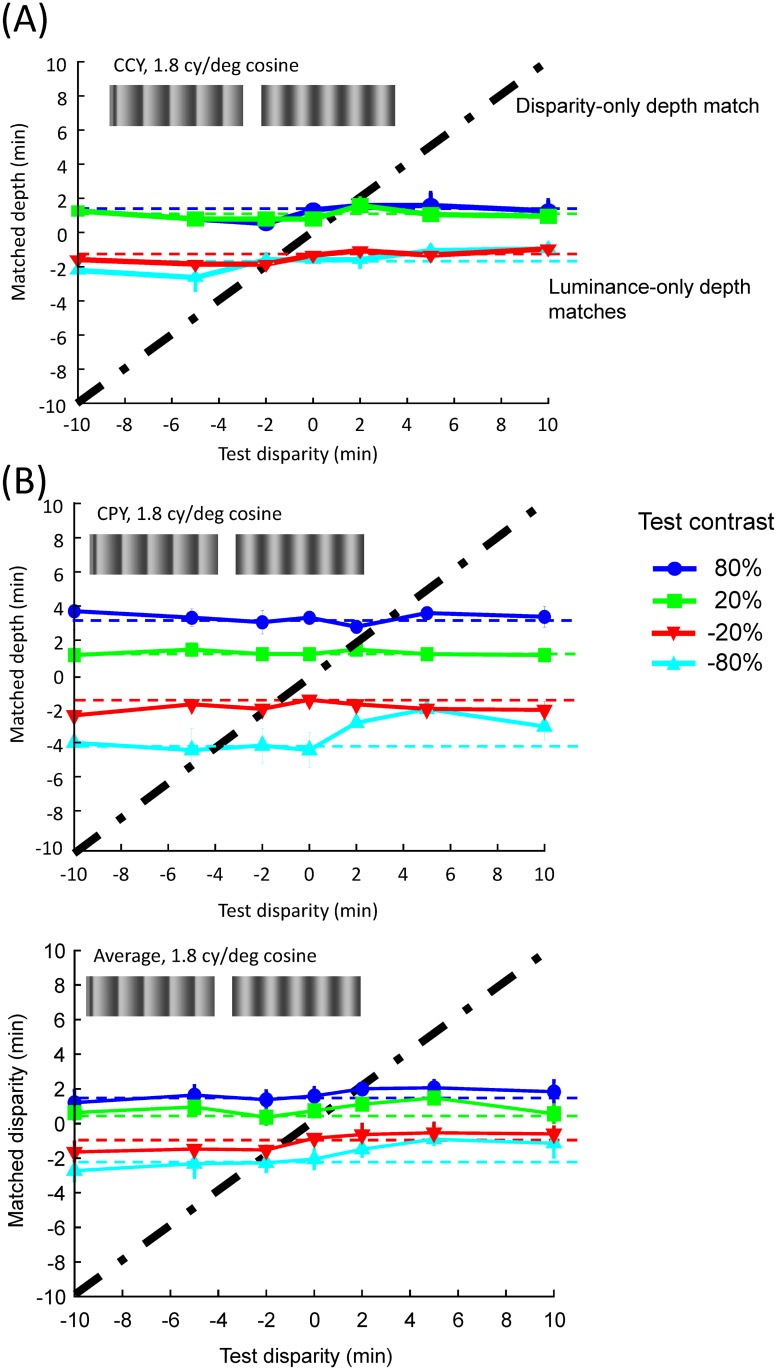
Matched depth as a function of the test disparity for luminance gradients in two representative observers (A) and (B), and the average across five observers (C) in the 1.8 *cy*/*deg* cosine wave condition. Plotting conventions are the same as Fig 2.

The first experiment was to match the perceived depth of the combined luminance and disparity targets as a function of test disparity. Fig 2 shows the matched disparity as a function of test disparity of the raised cosine stimuli (0.26 *cy*/*deg* test spatial frequency) at four test luminance contrasts for two representative observers, and the average across five observers. Fig 3 shows the same result for the 1.8 *cy*/*deg* cosine wave conditions. For individual data, each data point is the mean of at least four measurements. The error bars represent one standard error of the means. The horizontal dashed lines denote the matched disparity for the case of zero test disparity for the corresponding test luminance contrast. Since these matches were acquired without a disparity between the left and right eye images, the observer could only rely on their impression of depth from the luminance gradient in the image for the judgment. Hence, they represent the level of perceived depth from luminance contrast alone. The diagonal dash-dot lines show the predictions for depth matches by target disparity only. Thus, if the perceived depth of the target was determined by the disparity modulation alone, the matches made by the observers should all fall on this diagonal line.

For all conditions, the matched disparity was roughly invariant for each luminance contrast regardless of the magnitude and the sign of the test disparity, implying that the perceived depth modulation was determined by luminance cues alone. As a result, all the matched functions lie near the horizontal dashed lines defined by the match for the zero test disparity condition. To quantify this behavior, we fit a linear function with slope as a free parameter to each of the 40 test disparity functions (4 luminance contrasts × 2 spatial frequency × 5 observers). Only 7 out of 40 functions had slopes that were significantly different from 0 at the p < 0.05 level and none was significant when we applied Bonferroni correction to the statistical tests to control for multiple applications. Notice that, our result was that the matched disparity did not depend on disparity modulation in the test stimuli. It was not that the observer did not have a sense of depth from disparity. As the readers can see for themselves from binocular fusion of the stimulus examples in Fig 1, these stimuli do give a sense of disparity-induced depth. It is just that the perceived depth did not change with test disparity.

On the other hand, observers had no problem perceiving depth from the luminance modulation (again as demonstrated in Fig 1). Luminance increases were always perceived as convex (positive disparity match) and decreases as concave (negative disparity match). This is effect is most pronounced in observer CPY (Panel (A) in Figs 2 and 3). Even observers CCY (Panel (B) in Figs 2 and 3), who showed the weakest luminance contrast effect among all our observers, had a clear effect of contrast polarity. The data of all other observers and the mean of all five observers (Panel (C) in Figs 2 and 3) fall between the two extremes. These effects were confirmed by a three-factor (luminance contrast × test disparity × spatial frequency), nested (test disparity was nested to spatial frequency), repeated measures ANOVA, which showed a significant effect for luminance contrast (F(3,220) = 129.62, p < .0001) but not for test disparity (F(12,220) = 1.55, p = .11). Such result is consistent with the “dark-is-deep” rule for shape from shading in diffuse illumination [6,26,28–31] in the case of the cosine wave modulation. For the single raised cosine the interpretation is not so clear, as developed in the Discussion.

**Fig 4:**
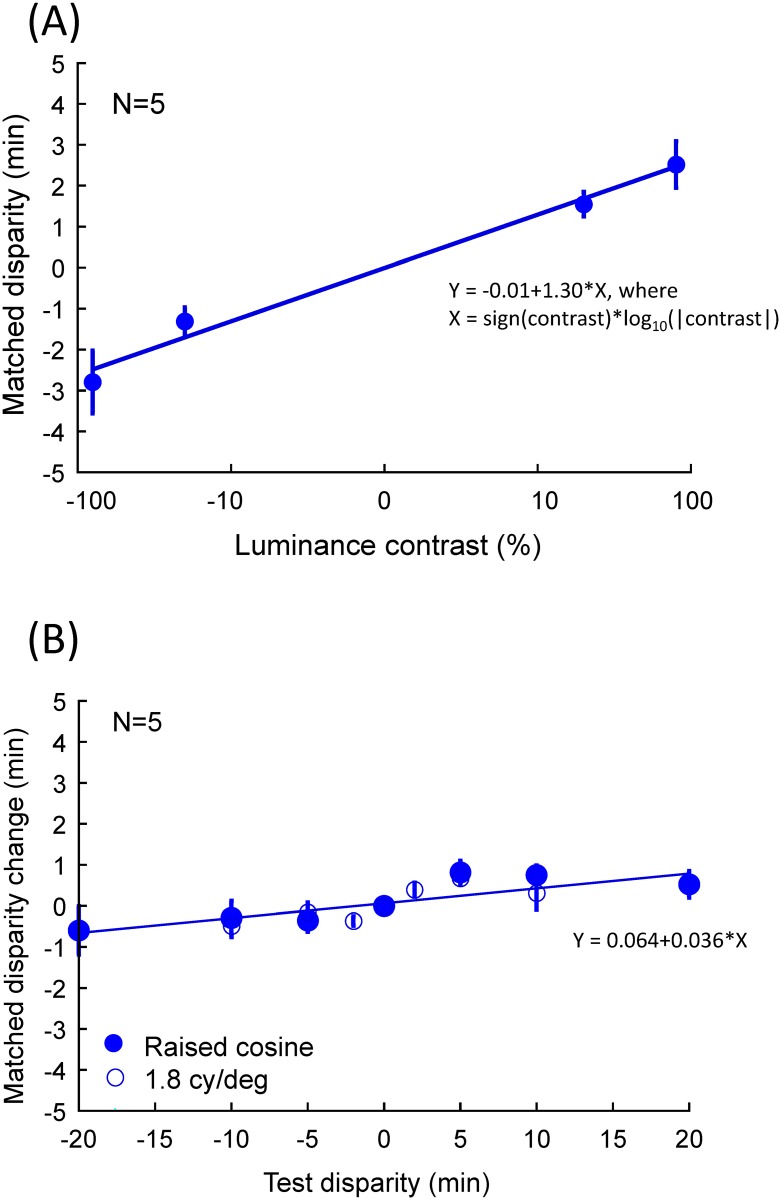
(A) Matched random-dot disparity as a function of luminance contrast at the zero-disparity conditions, averaged across observers. (B) Matched random-dot disparity as a function of test disparity for the luminance gradients, normalized by the perceived depth in the zero-disparity condition, averaged across luminance contrasts and observers. Solid circles are the raised cosine condition and open circles, the 1.8 *cy*/*deg* condition. Error bars represents one standard error of the means across observers.

To further illustrate the substantial effect of depth from luminance alone, Fig 4(A) shows the matched disparity as a function of luminance contrast, averaged across observers for the zero disparity conditions. On average, matched disparity was close to a linear function of log luminance contrast (over a factor of 4 in positive and negative luminance contrasts) and was best fit by the function

Y = −a + b X

where X = sign(*contrast*)×log_10_(|*contrast*|), with *a* = -0.01 arcmin and *b* = 1.30 arcmin/log_10_ unit which explains 98% of the variance in the averaged data at zero test disparity.

On the other hand, the change of matched disparity caused by the test disparity alone (Fig 4B) was remarkably flat ((slope *b* = 0.036 arcmin/log_10_ unit), implying that perceived depth for the disparity of the luminance gradients is less than 4% of that from the random-dot matching disparity modulation. Thus, the perceived depth of the dual-cue sinusoids was strongly dominated by the luminance modulation *per se* and received surprisingly little contribution from their disparity modulation.

### Phenomenology

**Fig 5:**
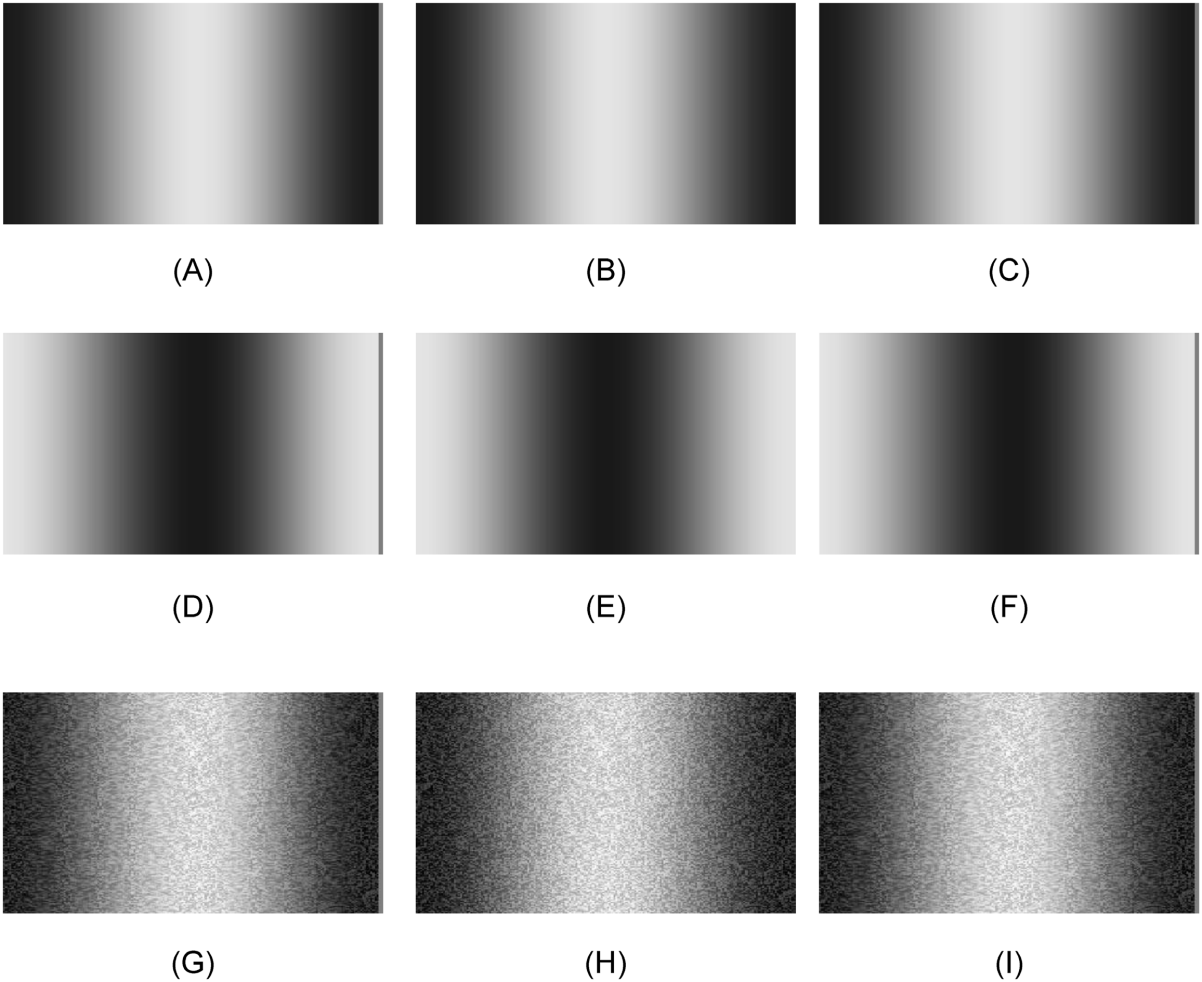
Demonstration of the luminance depth effect. (A) and (B) have the same positive raised cosine luminance and disparity modulation as the left and the right eye images of our stimuli (see Method). (C) is a copy of (A). Hence, binocular fusion of (A) and (B) should give a image that has an opposite disparity from fusion of (B) and (C). Yet, observers perceive a convex shape in both types of fusing. (D)-(F) are the same as (A)-(C) except that the luminance profile has the opposite polarity. Now the depth is perceived as concave in both cases, regardless of the direction of the disparity information. In Panels (G)-(H), the images of Panels (A)-(C) are replicated but random dots are added with the same disparity information as the luminance shifts, making the disparity-induced depth sufficiently strong to overcome the depth from the luminance cue that dominates in Panels (A)-(C).

The reader can observe such luminance dominance effect with illustration shown in Fig 5. In each image of the top row of Fig 5 (Panels (A)-(C)), the reader can readily perceive a convex shape with the center bulge toward the viewer. If one fuses Panels (A) and (B), the disparity between the two images gives a percept of stereo-depth. Panel (C) is exactly the same as Panel (A) but placed to the left of Panel (B) instead of to the right. Hence, the disparity between Panels (B) and (C) has the opposite polarity from that between Panels (A) and (B), so it should be seen as a concave shape bulging away from the viewer. However, the reader can verify that both pairings are always perceived as a convex bulges regardless of which pair of images is fused.

The middle row of Fig 5 (Panels (D)-(F)) is the same as the upper row except the luminance profile has the dark at the center. Thus, the viewer perceives a concave shape in both pairings, despite the opposite disparity signals in the two cases. Again, the disparity information plays little role in perceived shape, although a small difference may be perceived in the convexity in the upper row and the concavity in the middle row that is attributable to the disparity cue adding weakly to the luminance cue to perceived depth.

The bottom row of Fig 5 (Panels (G)-(I)) illustrates how the depth should be perceived from the disparity cue if it were fully processed. Panels (G)-(I) replicate the images of Panels (A)-(C) but overlaid with random dots having the same disparity information as the luminance shifts. This modification demonstrates that the random-dot overlay can make the disparity-induced depth sufficiently strong to overcome the depth from the luminance cue that dominates in Panels (A)-(C). However, the monocular cue of texture uniformity in the random dots provides a cue to flatness that does tend to reduce the maximum depth impression relative to that for the luminance cue alone.

## Discussion

In this study, we show that observers can perceive measurable depth from luminance gradients, with the brighter regions appearing closer to the observer than dark regions. Since our stimuli have the same luminance profile as a surface under diffuse illumination under the Lambertian reflectance assumption, this result confirms that observers can recover shape from shading under diffuse illumination on the basis of the “dark-is-deep” rule [6,28–31]. In addition, we confirm that the perceived depth increases in proportion to the logarithm of luminance contrast, although this was not a strong test because the range was only a factor of 4.

The interpretation of the “dark-is-deep” rule in terms of shape from shading under diffuse illumination (and assuming Lambertian reflectance) is strictly accurate only for the cosine wave, in the sense that a physical surface has the aperture to the ‘sky’ (or diffuse illumination source) for each point on the surface that varies appropriately to give a monotonic relationship between the surface intensity profile and the physical depth [28]. (Note that, under the usual shape-from-shading rule for a point source, both the peaks and troughs of a sinusoidal corrugation would appear brighter than the flanks when the source was normal to the mean surface, and hence the luminance modulation would have double the spatial frequency of the depth modulation. Conversely, if the point source were at a grazing angle to the flanks, the depth peaks would appear to be shifted to the halfway points between the luminance peaks and troughs. Neither of these point-source interpretations were perceived by the observers, who all reported the light peaks as closer, consistent with the diffuse illumination assumption.)

The similar result, of the light peak appearing the closest for the single raised cosine, can be seen as a generalization of the same perceptual interpretative principle, even though there is no illumination source (diffuse or local) that can generate a cosine intensity profile from a raised cosine surface. For symmetrical illumination, the far tails of the raised cosine should have the same intensity as the central peak under the Lambertian reflectance assumption, while the slanted flanks should appear dimmer. The fact that the human visual system shows essentially the same results for the two configurations (see Fig 4) implies that it is applying the shortcut heuristic suggested by Tyler [28] to determine the perceived depth, even though it is not entirely appropriate in the case of the single cosine target.

Remarkably, the binocular disparity cue in our test stimuli had little effect on the perceived depth. At the first glance, this result is inconsistent with the previous studies showing that the perceived depth of a surface is jointly determined by the disparity and shading cues [36,39]or that the sensitivity to the 3D shape variation of a surface from shading cue can be improved by the presence of stereoscopic cues [35,38], or that the perceived position of a surface is more strongly determined by disparity than by luminance cues to the shape [40]. There are three possible reasons underlying this inconsistency. First, all previous studies of shading cues compared the shading cues with disparity cues from added, local-edge-defined disparities rather than with disparity cues defined by the shading information, *per se*. In our study, the only disparity cue available is that provided by the intrinsic disparities of the shading gradients produced by the lighting of smooth surfaces. Such intrinsic ‘shading’ disparities are of the same (low) spatial frequency content as the surface gradients, which may not be optimal for the disparity processing system [43], and hence may not provide a reliable depth cue.

Second, all those studies showing an interaction with disparity and shading cues used shading under directional illumination. The computation of shape-from-shading under directional and diffuse illumination is quite different, and may even be processed by different mechanisms in the visual system [31]. Hence, the interaction between stereoscopic and shading cues may be different under directional and diffuse illumination. Third, it has been suggested that binocular disparity plays a secondary role in perceived 3D shape if there is a strong 2D shape cue, such as contour symmetry, in the stimuli [44]. For example, Norman et al. [45] reported that adding binocular disparity to the stimuli does not help shape discrimination threshold if there are enough monocular cues in the stimuli. That is, if there are strong monocular depth cues available, observers tend to make shape judgments based on the monocular cues to depth and ignore disparity information. Our stimuli were designed to simulate the binocular view of smooth surfaces under diffuse illumination. Given that diffuse illumination is the default assumption for illumination used by human observers [28], it is likely that the shading cues in our stimuli were much stronger than the binocular cues.

### Maximum Likelihood Analysis

The latter explanation of the lack of the disparity effect may be further analyzed in the context of Maximum Likelihood Estimation theory (MLE) for depth cue combination [46]. MLE theory suggests that the depth estimated from combined cues is a weighted average of the depths for the individual cues and that the contribution of each individual cue to the depth computation is proportional to the reliability of that cue (defined as the inverse of the variance of depth estimation). Formally, therefore, the perceived depth *d*_*p*_ is given as

$$d_{p\;=\;}w_{L}d_{L}+w_{D}d_{D}$$

where

$$w_{L}\;=\;\sigma_{D}^{2}/(\sigma_{D}^{2}+\sigma_{L}^{2})\text{ and }w_{D}\;=\;\sigma_{L}^{2}/(\sigma_{L}^{2}+\sigma_{D}^{2})$$

where *d*_*L*_ and *d*_*D*_ are the perceived depth induced by luminance and disparity cue, respectively, the weighting factors *w*_*L*_ and *w*_*D*_ are determined by the variances for the luminance cue, $\sigma_{L}^{2}$, and disparity cue, $\sigma_{D}^{2}$, respectively. Hence, the estimated depth of the combined cue should be a linear combination of the estimated depths of individual cues (assuming constant additive noise sources). In extreme cases where one cue is very reliable while the other provides no reliable depth information, the MLE predicts that the weighting effectively devolves to the reliable cue.

Qualitatively, the fact that the measured disparity functions are almost flat over most of the range (Fig 2) implies that our results are near the extreme case in which the observers maximized the luminance cue and heavily discounted the disparity cue. Hence, under MLE, they would imply either that the visual mechanisms for disparity are extremely noisy or that they contribute little to the perceived depth, neither of which seems consistent with the previous literature implying a much more accurate contribution to shape from disparity than from luminance information (e.g., Likova & Tyler,[40]).

For a quantitative test of the MLE theory, we used the matched disparity at the zero test disparity conditions (i.e., the central data points in each panel of Fig 2 for a given observer) as an estimation of the perceived depth induced by the luminance cue alone, *d*_*L*_, and the measured variance for the zero disparity conditions (averaged across different luminance contrast conditions for a given observer) as an estimation of the variance $\sigma_{L}^{2}$ for that observer. The variance of the non-zero test disparity conditions, $\sigma_{T}^{2}$, estimated by the measured variance averaged across the data for all non-zero test disparities, should contain contributions from both luminance and disparity. Hence, assuming that the noise source for the luminance and the disparity cues are independent, the variance for the disparity cue is computed as

$$\sigma_{T}^{2}\;=\;\sigma_{D}^{2}+\sigma_{L}^{2}$$

We understand that there is an alternative approach to estimate the variance of perceived depth with a separate just-noticeable difference (JND) measurement [47]. However, the variance in matching tasks we used is not only a more direct estimate but also has been used to estimate the JND in some classic psychophysics studies (e.g., MacAdam, [48]) and thus should provide a good estimation of the variance of perceived depth from different cues.

There was no independent measure of perceived depth from disparity alone in our data (since the disparities were, by definition, specified by the luminance gradients). However, since the range of our test disparities was relatively small (up to only about 1/10^*th*^ of the full range for depth from disparity), we can assume that the perceived depth is a linear function of test disparity, that is, *d*_*D*_ = *ad*_*t*_, where *d*_*t*_ is the test disparity in the experiment and *a* is the disparity scaling factor. For the noise cues, we assumed that the noise was additive and thus was at the same level for all conditions, and that the observers was limited by noise from both sources in all conditions and thus the weighting *W*_*L*_ and *W*_*D*_ were the same for all conditions. The MLE model for the perceived depth from shading gradients may thus be implemented with only *a* as a free parameter. This approach was compared with a no-free-parameter model of the depth for the non-zero disparity conditions being determined by the perceived depth from the luminance-only condition.

**Fig 6:**
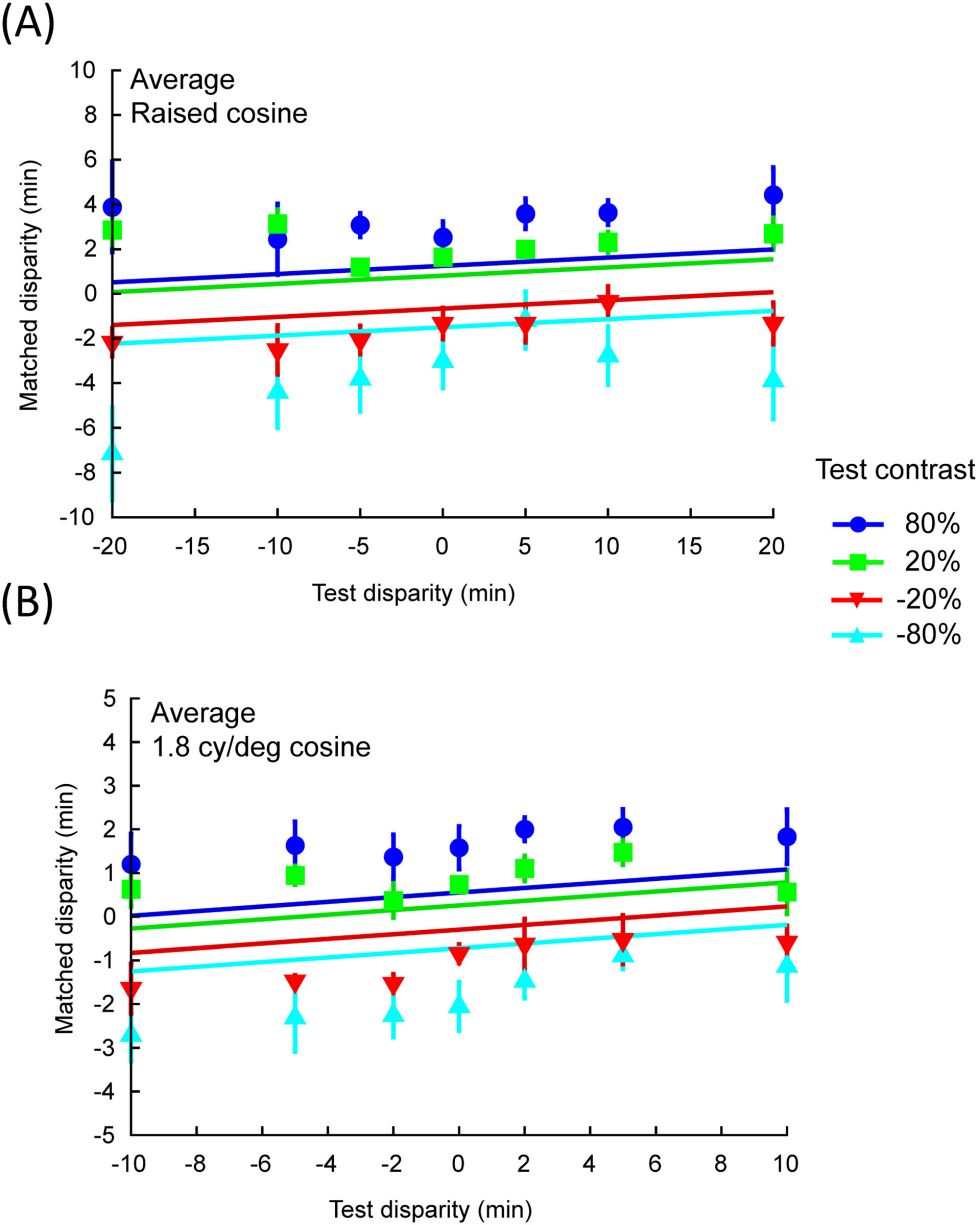
Example of the fit of the MLE model to the average data from Fig 2C (A) and Fig 3C (B).

Fig 6 shows an example of the fit of the 1-parameter MLE model to the data of Figs 2C and 3C. As this fit shows, in general the MLE model underestimates the effect of the luminance cue, in that the fitted curves are somewhat closer to the zero depth than the data points. The scale parameter required to fit the data, averaged across all observers, was about 0.26. That is, the variation in perceived depth according to the matched disparity was only a quarter of that of the physical disparity of the luminance gradients! The SSE of the MLE fit, with one free parameter for each observer and test spatial frequency, was about twice that of the fit of a horizontal line at the mean level for each luminance contrast and observer (zero free parameter fit). Hence, it is clear that the MLE theory of cue combination provides only a poor fit to the data.

The failure of the MLE theory to fit our data is inconsistent with the result of Lovell et al. [39] who showed that the MLE theory could account for the perceived depth provided by the combined disparity and point-source shape-from-shading cues. However, in their experiments, the luminance gradient for shading cues carried no disparity information. Instead, the disparity cue was provided by random dots whose luminance distribution was inconsistent with the shading cues. Thus, the shading cue was likely to have been degraded by the presence of the uniform random dot texture. Furthermore, the shading cues they used were created with Brewsterian directional illumination, whereas our stimuli were constructed to simulate diffused illumination. The discrepancy between their and our results is consistent with the notion that the shape-from-shading for a directional light source and for a diffuse light source are processed by different modules in the visual system [30].

## Conclusion

We show that the perceived depth modulation on a diffusely illuminated surface is consistent with the prediction of an increase in proportion with the logarithm of luminance contrast. Perceived concaveness of the depth modulation depended on luminance phase but was essentially unaffected by the luminance disparity modulation. The observers’ performance was consistent with what one expected from the dark-is-deep rule for perceived depth from shading under diffuse illumination. Thus, these results validate the idea that human observers can use the diffuse illumination assumption to perceive depth from luminance gradients alone without making the assumption of light direction. Moreover, when combined with the disparity cues for the same surface structure, the observers weighted luminance shading over the disparity information for depth judgments. This result cannot be explained by the maximum likelihood theory of cue combination.
